# Predicted Versus Non-Predicted Opioid Administration Using Preoperative Pain Sensitivity in Patients Undergoing Gynecological Surgery: A Randomized-Controlled Trial

**DOI:** 10.3390/jcm10040585

**Published:** 2021-02-04

**Authors:** Sun-Kyung Park, Hansol Kim, Seokha Yoo, Won Ho Kim, Young-Jin Lim, Jin-Tae Kim

**Affiliations:** Department of Anesthesiology and Pain Medicine, Seoul National University Hospital, Seoul National University College of Medicine, Seoul 03080, Korea; mayskpark@gmail.com (S.-K.P.); hansolfrkr@gmail.com (H.K.); muroki22@gmail.com (S.Y.); wonhokim.ane@gmail.com (W.H.K.); limyjin@snu.ac.kr (Y.-J.L.)

**Keywords:** postoperative pain, opioid, intravenous patient-controlled analgesia, nausea

## Abstract

Individualized administration of opioids based on preoperative pain sensitivity may improve postoperative pain profiles. This study aimed to examine whether a predicted administration of opioids could reduce opioid-related adverse effects after gynecological surgery. Patients were randomized to the predicted group or control group. Participants received a preoperative sensory test to measure pressure pain thresholds. Patients were treated with a higher or lower (15 or 10 μg/mL) dose of fentanyl via intravenous patient-controlled analgesia. The opioid dose was determined according to pain sensitivity in the predicted group, while it was determined regardless of pain sensitivity in the control group. The primary outcome was the incidence of nausea over the first 48 h postoperative period. Secondary outcomes included postoperative pain scores and opioid requirements. There was no difference in the incidence of nausea (40.0% vs. 52.5% in predicted and control groups, respectively; *p* = 0.191) and postoperative pain scores (3.3 vs. 3.5 in predicted and control groups, respectively; *p* = 0.691). However, opioid consumptions were lower in the predicted group compared to the control group (median 406.0 vs. 526.5 μg; *p* = 0.042). This study showed that offering a predicted dose of opioids according to pain sensitivity did not affect the incidence of nausea and pain scores.

## 1. Introduction

The treatment of postoperative pain is an ongoing challenge for anesthesiologists, despite the introduction of modern multimodal analgesia [1,2,3]. Postoperative pain has major adverse influence on patient recovery after gynecological surgery and is associated with a higher risk of complications and longer hospital stays [4]. Although opioids remain a cornerstone for postoperative pain management [1,3,5,6], opioid use is associated with undesirable effects including postoperative nausea and vomiting (PONV) [4,6,7,8,9,10]. In particular, gynecological surgery is associated with an increased risk of PONV, of which the incidence was reported in 26–57% of women [11,12,13,14].

Thus, optimal dosing of opioids after gynecological surgery requires offsetting the desired effects against the potential undesirable effects [3,15]. However, the optimization of opioid dosing is challenging because the experience of pain and response to analgesics are highly variable among individuals [3,4]. As a possible explanation for the inter-individual variability, pain sensitivity remains the most important factor [3,16,17]. Numerous studies have sought to develop a predictive tool for postoperative pain severity and many quantitative sensory tests are correlated with postoperative pain intensity [2,18,19,20]. Hsu et al. suggested that the assessment of preoperative pressure pain with a pressure algometer could predict postoperative pain intensity and analgesic consumption after gynecological surgery [20]. However, to date, no report exists on the clinical outcomes when these prediction modalities are used for administration of postoperative analgesic medications.

Therefore, this study aimed to determine whether a predicted administration of opioids, based on preoperative pain sensitivity, could reduce opioid consumption, drug-related side effects, and improve pain scores after gynecological surgery. We hypothesized that a predictive approach would reduce unnecessary opioid medications and opioid-related side effects. To test these hypotheses, we examined the postoperative analgesic properties and potential adverse effects between predicted and non-predicted administrations of postoperative opioids in patients receiving gynecological surgery.

## 2. Materials and Methods

### 2.1. Study Design and Participants

This prospective, randomized, double-blind, single-center trial was approved by the institutional review board of Seoul National University Hospital (No. 1707-078-869) and was registered with ClinicalTrials.gov (identifier NCT03252977; principal investigator: Jin-Tae Kim; date of registration: 14 August 2017). This study adheres to the Consolidated Standards of Reporting Trials (CONSORT) guidelines [21]. All patients provided written informed consent. The study was conducted between August 2017 and November 2017 at Seoul National University Hospital, Seoul, Korea.

This study included adult patients with American Society of Anesthesiologists physical status I/II/III, who were scheduled to undergo elective gynecological surgery under general anesthesia. Eligible surgeries included laparoscopic ovarian cystectomy, laparoscopic salpingo-oophorectomy, laparoscopic hysterectomy, abdominal hysterectomy, abdominal myomectomy, and vaginal hysterectomy. Patients with contraindications to receiving fentanyl, chronic pain, preoperative use of any chronic pain medications, such as opioids, nonopioid or nonsteroidal anti-inflammatory drugs, renal insufficiency, morbid cardiovascular impairments, body weight <40 kg or >90 kg, or pregnancy, were excluded from the study.

### 2.2. Preoperative Assessment of Pain Sensitivity

**Figure 1:**
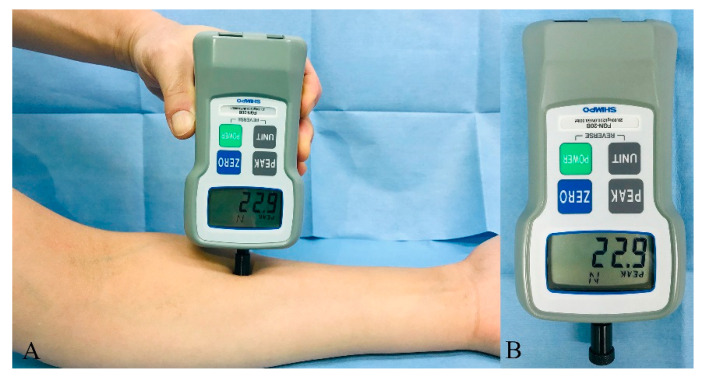
Quantitative sensory test to determine the pressure pain threshold of each patient. (**A**) The pressure was increased until the patient felt pain. (**B**) The pressure at which each patient started to feel pain was measured and recorded using the pressure algometer.

All patients underwent quantitative sensory tests using an electronic handheld pressure algometer (Digital Force Gauge FGN-20B, Shimpo Corp., Kyoto, Japan) to determine the pressure pain thresholds the day before surgery. Briefly, a flat probe (diameter, 12 mm) was applied to the left forearm and the pressure was increased at a speed of 2 N/s (Figure 1A). Patients were asked to raise their right hand when they felt pain (pain threshold) and the algometer recorded the pressure at that time (Figure 1B). The sensory test was repeated three times at a time interval of 10 minutes. The mean pressure threshold was recorded as the baseline pressure pain threshold for each patient. The cut-off value of the pressure pain threshold for dividing patients into sensitive and non-sensitive patients was determined before commencing the trial from preliminary data acquired at our institution and the previous literature [22,23]. Two previous studies reported a mean pressure pain threshold of 24.9 and 26.7 N in 54 patients [22] and 10 volunteers [23], respectively. A pilot study at our institution found a mean pressure pain threshold of 25.8 N in gynecological patients. Thus, we used 26 N as a cut-off threshold in this study. Patients with a threshold of <26 N were considered sensitive patients (i.e., patients with a higher risk for severe postoperative pain). Patients with a threshold ≥26 N were considered non-sensitive patients (i.e., patients with lower risk for severe postoperative pain).

### 2.3. Randomization and Minimization of Bias

The study involves two potential regimens (higher- or lower-dose) of intravenous (IV) fentanyl via patient-controlled analgesia (PCA) for postoperative pain management. The higher- and lower-dose regimens were fentanyl 15 and 10 μg/mL, respectively. Both regimens included nefopam 0.8 mg/mL. These are the standard regimens of IV PCA after gynecological surgery in our institution. Patients were randomly assigned to the predicted or non-predicted regimen using a computer-generated table of random numbers. In predicted group, sensitive and non-sensitive patients used the higher- and lower-dose regimens, respectively. In the non-predicted (control) group, patients used one of the two regimens, regardless of their own pain threshold, by pairing to the predicted group. Group allocation was concealed using a sequentially numbered, sealed opaque envelope, which was opened only by a separate investigator and delivered to the nurse who prepared the IV PCA. The investigator and nurse had no further involvement with the patients and every IV PCA had an identical appearance; thus, participants, anesthesiologists, and outcome assessors were blinded to group allocation. The gynecological surgeons and nurses were blinded to the study protocol.

### 2.4. Anesthetic Procedures

All patients received the following standardized anesthetic managements. No premedication was administered before surgery. Upon arrival in the operating room, standard monitoring was applied. General anesthesia was induced and maintained by target-controlled infusion of propofol and remifentanil. The bispectral index was used to monitor the depth of anesthesia. After administration of 0.8 mg/kg rocuronium, tracheal intubation was performed. During the operation, the bispectral index was maintained between 40 and 60. Remifentanil was titrated to maintain systolic arterial pressures between 100 and 140 mmHg throughout the surgery. Local anesthetic was not administered for skin closure. The amount of intraoperative remifentanil used was recorded. No opioids medications other than remifentanil were administered during surgery.

An IV PCA pump (AutoMed 3200, Ace Medical, Seoul, Korea) was connected at the end of surgery. The IV PCA was set to deliver 1 mL bolus with a 15 min lockout time, and 1 mL/h background infusion. All patients received IV ramosetron 0.3 mg before the start of the IV PCA for prophylaxis against postoperative nausea and vomiting (PONV).

### 2.5. Postoperative Management Protocol

After the surgery, patients were transferred to the post-anesthesia care unit (PACU). Patients were discharged from the PACU after a score of 10 was obtained on the 14-point modified Aldrete’s scoring system by a blinded PACU nurse.

Standardized postoperative analgesia was provided to every patient until hospital discharge. IV PCA was maintained for the first 48 h postoperative period in principle, and in the case of discontinuation before the 48 h, the timing and reasons were recorded. IV ketorolac (30 mg) was used as a rescue analgesic for breakthrough pain as required for a numerical rating scale (NRS) ≥4 during the postoperative 3-day periods. Regular oral analgesics, including 80 mg of zaltoprofen every 8 hours, were administered on postoperative days 1 and 2. IV ramosetron (0.3 mg) was administrated for PONV prophylaxis on postoperative day 1. If PONV occurred, 10 mg of IV metoclopramide was administered as a rescue antiemetic.

### 2.6. Outcome Assessments

The primary outcome was the incidence of postoperative nausea during the 48 h study period. Secondary outcome variables included postoperative pain scores at 3, 24, and 48 h after the completion of surgery, cumulative fentanyl consumption via IV PCA within the first 3 h, 3–24 h, and 24–48 h postoperatively, rescue analgesic consumption within the 48 h study period, opioid-induced adverse events, including vomiting, pruritus, shivering, respiratory depression, and urinary retention, and patient’s overall satisfaction scores with postoperative pain management (0 = totally unsatisfied and 100 = totally satisfied) for the first 48 h postoperatively. Cumulative nefopam consumptions within the first 3 h, 3–24 h, and 24–48 h were also analyzed.

Regular postoperative follow-up assessments were conducted at 3, 24, and 48 h after the completion of surgery by a blinded investigator. Pain intensity was recorded on the verbal NRS from 0 to 10. The requirements of rescue analgesics and cumulative dose of rescue analgesics were recorded at 3, 24, and 48 h after the end of surgery. The occurrence of postoperative nausea, vomiting, and urinary retention was recorded. In patients who experienced nausea, the severity of nausea was rated on the NRS (0 = none and 10 = worst imaginable).

### 2.7. Statistical Analysis

Data were analyzed using SPSS Statistics for Windows (version 23.0, IBM Corp., Armonk, NY, USA) and R software (version 3.6.2, R Foundation for Statistical Computing, Vienna, Austria). All data were analyzed on an intention-to-treat basis. Categorical data were expressed as a number (proportion with 95% confidence interval) and analyzed using the Pearson χ^2^ test. Categorical variables were analyzed using Fisher’s exact test when the expected counts were < 5 for at least 25% of the cells. Intergroup differences in the incidence of postoperative nausea during the 48 hours study period, the primary outcome, were assessed for significance using the Pearson χ^2^ test. Continuous data were tested for normality using the Shapiro–Wilk test. Data showing a normal distribution were presented as mean (SD), and data showing a non-normal distribution were presented as median (interquartile range). Continuous variables were analyzed using Student’s *t* test or Mann–Whitney *U* test for intergroup differences, as appropriate. Pain scores and the amount of fentanyl consumptions were considered continuous variables. Two-tailed *P* values < 0.05 were considered statistically significant. Repeated measurements (pain scores and amount of fentanyl consumptions) were analyzed using the Mann–Whitney *U* test with Bonferroni correction. The criterion for rejection of the null hypothesis for the repeated measurement at each time point was *p* < 0.017. Pre-specified subgroup analyses included the sensitivity to pressure pain.

### 2.8. Sample Size

Sample size was calculated based on preliminary data obtained in the patients undergoing gynecological surgery at our institution. The observed incidence of nausea after gynecological surgery was 45%. We hypothesized that the use of a predicted regimen of opioids could reduce the incidence of postoperative nausea to 20%. To achieve a power of 0.8 and an alpha error of <0.05, 54 patients were required in each group. To allow for dropout, 59 patients were randomized to each group.

## 3. Results

**Figure 2:**
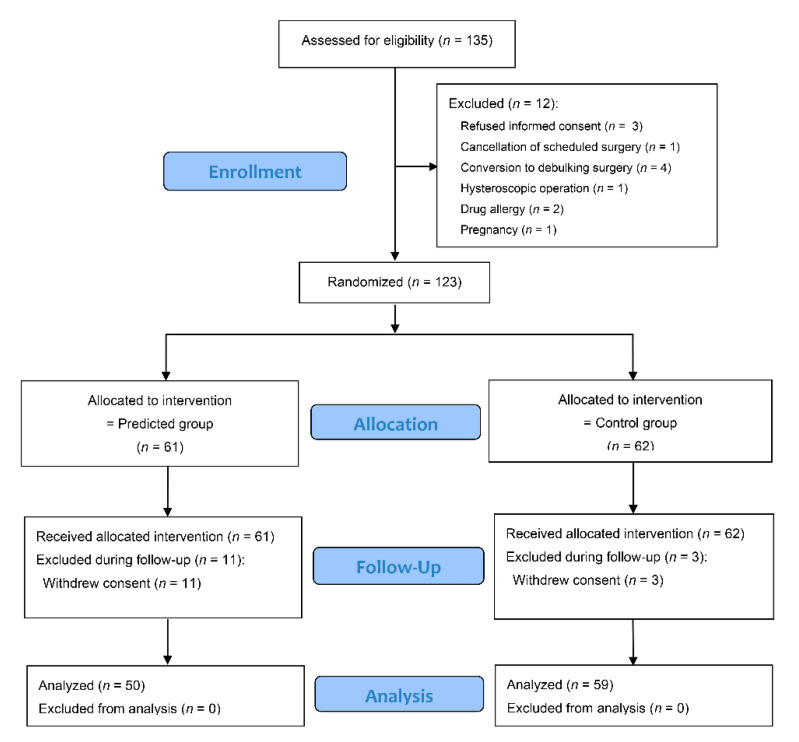
CONSORT flow diagram of patient recruitment. CONSORT indicates Consolidated Standards of Reporting Trials.

**Figure 3:**
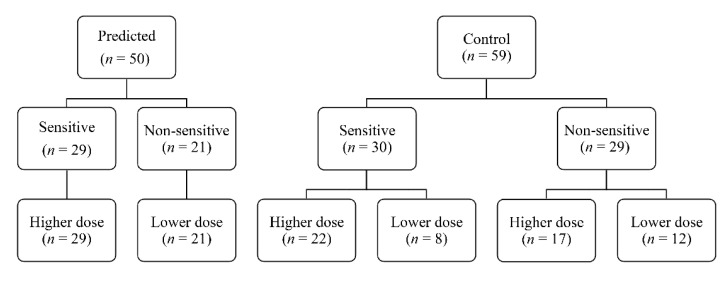
Flow diagram showing a breakdown of the randomization, pain sensitivity, and administered analgesic regimens of study populations.

Of 123 patients who underwent randomization, 14 patients were excluded because of withdrawal of patient consent; therefore, 109 subjects completed the study (Figure 2). The baseline characteristics were comparable between groups (Table 1). All subjects were non-smokers, and no patient had a history of motion sickness or PONV. As a result, all subjects had three risk factors for PONV (female gender, non-smoker, and postoperative use of opioids) according to the Apfel score [24]. The mean pressure pain thresholds were 28.2 N and 28.6 N in the predicted and control groups, respectively. Figure 3 shows a breakdown of group allocations, preoperative pain sensitivity, and the IV PCA regimens per group.

### 3.1. Opioid Use, Pain Scores and Opioid-Related Side Effects in Predicted versus Non-Predicted Groups

Providing patients with a predicted dose of fentanyl according to their preoperative pain sensitivity significantly reduced the opioid consumption via IV PCA during the first 48 h postoperative period (median, 406 µg vs. 526.5 µg in the predicted vs. control groups, respectively; difference of median, −121 µg (95% CI, −279 to −17 μg); *p* = 0.042; Table 2). However, offering patients the predicted dose according to their pain sensitivity did not reduce the incidence of nausea during the first 48 h postoperative period (40% vs. 53% in the predicted vs. control groups, respectively; relative risk, 0.8 (95% CI, 0.5–1.2); *p* = 0.191; Table 3). Pain scores showed no significant differences between groups (mean = 3.3 vs. 3.5 in the predicted vs. control groups, respectively; *p* = 0.691; Table 2). Nefopam consumptions showed no significant differences between the two groups (Table 2). Sixteen patients discontinued IV PCA during the first 48 h postoperative period (Table 2). The reasons for the discontinuation of IV PCA were as follows: nausea (three patients in each group), dizziness (two patients in control group), tolerable pain (three patients in the predicted group), swelling around intravenous catheter insertion site (one patient in control group), and preparation for early discharge (two patients in each group). There was no difference in the rescue analgesic requirements and the incidences of other adverse events between groups (Table 2 and Table 3).

### 3.2. Effect of the Opioid Dose Given to Sensitive Patients

We conducted pre-specified subgroup analyses according to patient’s pain sensitivity. Fifty-nine patients were considered as sensitive patients (pain threshold <26 N; mean threshold 21.3 N). Among these patients, 51 patients were administered with the higher dose regimen, which corresponded to their sensitivity, while eight patients were administered with the lower dose regimen. The pain scores, fentanyl consumption, nefopam consumption, and incidences of postoperative nausea at each recorded time point were not significantly different between the patients receiving a higher or lower dose (Supplemental Table S1).

### 3.3. Effect of the Opioid Dose Given to Non-Sensitive Patients

Fifty patients exhibited a threshold ≥26 N and were considered as non-sensitive patients (mean threshold 36.8 N). We treated 17 patients with a higher dose regimen, which did not correspond to their sensitivity, and 33 patients with a lower dose regimen, which corresponded to their sensitivity. The incidence of nausea was significantly lower in patients treated with a lower dose regimen compared with a higher dose regimen (27.3% vs. 64.7%; *p* = 0.015; Supplemental Table S1). The pain scores during the 48 h study period showed no significant differences according to the regimen administered (3.7 vs. 3.3 for the higher vs. lower dose, respectively; *p* = 0.498; Supplemental Table S1).

### 3.4. Exploratory Analyses

We performed the exploratory subgroup analyses as per the types of surgery to address the potential confounding effects of the different surgical stresses. Most of the patients (70/109, 64%) received laparoscopic surgery, and median fentanyl consumption for the 48 h study period showed no statistically significant difference between the groups among these patients (341 µg vs. 468 µg for predicted group vs. control group, respectively; *p* = 0.060; Supplemental Table S2). We found no inter-group differences in pain scores, incidence of nausea, and vomiting, and nefopam consumption, as the overall analysis.

## 4. Discussion

In the present study, we examined the role of a predicted administration of opioids, based on preoperative pain sensitivity, in women undergoing gynecological surgery. Offering a predicted regimen of opioids based on the pressure pain threshold of individual patients reduced opioid consumption without increasing postoperative pain scores; however, it did not affect the incidence of opioid-related complications following gynecological surgery.

In recent years, many studies have investigated whether preoperative pain sensitivity of a patient can predict postoperative pain intensity and opioid requirements [2,20,25,26]. Hsu et al. demonstrated that the postoperative pain score and morphine consumption were correlated with preoperative pressure pain sensitivity assessed by algometer in women undergoing gynecological surgery [20]. Brandsborg et al. showed that decreased cutaneous and vaginal pressure pain thresholds before surgery were correlated with the intensity of early postoperative pain following hysterectomy [25]. Abrishami et al. reported that pain sensitivity, as measured by a preoperative sensory test, was correlated with postoperative pain intensity in women undergoing obstetric/gynecological procedures [2]. Thus, we conducted this study to determine whether the adoption of such predictive quantitative sensory tests in postoperative pain managements can improve clinical outcomes in women undergoing gynecological procedures. As previous evidence suggested that pressure pain threshold is correlated with postoperative pain intensity after gynecological surgery [20], we used the pressure pain threshold as a determinant of pain sensitivity in the current trial.

To the best of our knowledge, this was the first clinical study evaluating the utility of a predictive quantitative sensory test for postoperative pain managements in clinical practice. The conventional “one-size-fits-all” administration of analgesic medications frequently results in unnecessarily overdosing patients who are less sensitive to pain, while insufficient analgesia in patients who are sensitive to pain [3,15]. Therefore, preoperative identification of patients who have an increased risk of severe pain has been postulated to have great benefits in the postoperative setting [25]. We hypothesized that an individualized approach based on the patient’s own pain sensitivity could improve analgesic profiles and reduce opioid-related side effects compared with the conventional approach. However, our results showed no clinically relevant benefit in the predicted group with respect to pain scores and opioid-related side effects. The subgroup analyses for non-sensitive patients showed a potential benefit of an individualized approach by reducing the incidence of nausea in patients who were treated with a lower dose compared with a higher dose, although these results were not sufficiently powered. Nonetheless, to move forward in the context of individualized medicine, the utility of the predicted approach should be examined in the clinical setting of postoperative pain management.

Our choice of primary outcome needs discussion. When planning this trial, we hypothesized that a predicted administration of opioids based on the pain sensitivity could reduce the incidence of drug-related adverse events by avoiding unnecessary medications in patients with a low risk of severe pain. The primary endpoint of the current study was chosen based on its importance to patient satisfaction and the clinical relevance. The incidence of nausea was set as the primary outcome measure of this study because we considered nausea as the most common and distressing complication relating to opioids in women undergoing gynecological surgery. However, of note, the incidence of nausea during the 48 hours postoperative period may not be the most appropriate endpoint for determining the effectiveness of the use of opioids according to pain sensitivity of individual patients. It cannot be excluded that using a different primary outcome (e.g., pain scores or opioid consumption) or co-primary outcomes might have altered the results.

The non-predicted administration strategy in this trial also requires discussion. We measured pain sensitivity in both groups to blind all participants to group allocations. In the control group, we used the regimen paired with the administered regimen in predicted groups to balance the administered opioid regimens between the intervention and control groups. We did not use a deliberately un-matched regimen to patient’s sensitivity because we considered that this would not occur in a clinical setting; therefore, it would not reflect real practice. Moreover, we considered this to be potentially unethical. As a result, 58% of patients in the control group were treated with a regimen that corresponded to their sensitivity in this study. Thus, even if there are some beneficial effects of the predicted approach, these might not be detected in the current trial.

The following limitations should be considered when interpreting the results of our study and these limitations may suggest some implications for future investigations. First, we used only the threshold to pressure pain in this study. Other measurements of preoperative pain sensitivity, including threshold or tolerance to thermal or electrical stimuli, may lead to different results. Second, although the cut-off value to divide patients into the sensitive or non-sensitive patients was determined by a preliminary study and the previous literature, the nature of this sensitivity was not discrete but continuous. Third, we could not record the number of bolus administrations of PCA since the PCA pump used in this study had no record of the number of bolus given. Fourth, we had set the study follow-up period as 3 days postoperatively, but we changed it to 48 h postoperatively, for consistency between patients, before commencing the trial. However, the changes were not adequately reflected in our registry protocol, and it should be considered when interpreting our data. Fifth, we did not determine the regimen based on the patients’ body weight. Lastly, a relatively large proportion (58%) of patients in the control group received the regimen correctly matched to their sensitivity; therefore, the results of this study should be interpreted cautiously. Further research with better design and sufficient sample size is required on this subject.

## 5. Conclusions

This study failed to observe clinically relevant benefits of a predicted administration of opioids based on preoperative pain sensitivity for postoperative analgesic profiles in women undergoing gynecological surgery. However, offering a predicted dose of opioids according to a preoperative pressure pain threshold of each patient did lead to reduction in opioid consumption in the 48 h postoperative period, without a difference in pain scores and drug-related side effects between two groups. Further studies are required to determine whether individualized pain treatment protocols based on the patient’s own pain sensitivity improve analgesic profiles compared to “one-size fits all” protocols in postoperative settings.

## Figures and Tables

**Table 1:** Demographic and surgical data.

Characteristics	Predicted Group (*n* = 50)	Control Group (*n* = 59)	*p* Value
Age (y)	48.7 (14.5)	48.6 (13.3)	0.995
Weight (kg)	59.1 (9.1)	59.5 (8.8)	0.840
Height (cm)	157.3 (6.4)	158.5 (5.1)	0.272
Body mass index (kg/m^2^)	23.9 (3.5)	23.7 (3.3)	0.723
ASA physical status (I/II)	37/13 (74%/26%)	52/7 (88%/12%)	0.082
Type of gynecological surgery			0.611
Laparoscopic	30 (60%)	40 (68%)	
Abdominal	11 (22%)	12 (20%)	
Vaginal	9 (18%)	7 (12%)	
Operative time (min)	93 (54–151)	95 (65–145)	0.692
Anesthesia time (min)	130 (85–185)	135 (105–175)	0.293
Intraoperative remifentanil use (µg)	725 (500–1000)	1000 (550–1000)	0.063
Pressure pain threshold (N)	28.2 (9.2)	28.6 (9.8)	0.857
Sensitive/Non-sensitive	29/21 (58%/42%)	30/29 (51%/49%)	0.455
Higher dose/Lower dose	29/21 (58%/42%)	39/20 (66%/34%)	0.502
Use of the correspond regimen to sensitivity	50 (100%)	34 (58%)	< 0.0001
Hospital stays (d)	4 (3–5)	4 (3–5)	0.905

Data presented as mean (standard deviation) or median (interquartile range) or number of patients (proportion). Abbreviations: ASA, American Society of Anesthesiologists.

**Table 2:** Postoperative pain scores and analgesic consumptions.

Outcomes	Predicted Group (*n* = 50)	Control Group (*n* = 59)	*p* Value
Pain score at 3 h postoperatively (NRS)	5 (2–7)	5 (2–7)	0.946
Pain score at 24 h postoperatively (NRS)	3 (2–4.3)	3 (2–5)	0.232
Pain score at 48 h postoperatively (NRS)	3 (2–3.3)	2 (1–4)	0.491
Mean pain score, during the 48 h postoperative period (NRS)	3.3 (1.8)	3.5 (1.8)	0.691
Fentanyl consumption via IV PCA, within the first 3 h postoperatively (µg)	68.5 (45–117.8)	88 (60–119)	0.166
Fentanyl consumption via IV PCA, 3–24 h postoperatively (µg)	214.8 (145.5–294.8)	264 (152–352.5)	0.170
Fentanyl consumption via IV PCA, 24–48 h postoperatively (µg)	64 (0–246.8)	222 (0–307.5)	0.089
Total cumulative fentanyl consumption via IV PCA, for the first 48 h period (µg)	406 (309.8–614.6)	526.5 (370.5–718.5)	0.042
Nefopam consumption via IV PCA, within the first 3 h postoperatively (mg)	4.6 (3.0–6.6)	5.0 (4.0–7.5)	0.203
Nefopam consumption via IV PCA, 3–24 h postoperatively (mg)	14.4 (8.9–17.8)	14.6 (11.3–20.0)	0.401
Nefopam consumption via IV PCA, 24–48 h postoperatively (mg)	5.1 (0.0–18.2)	14.3 (0.0–19.1)	0.157
Total cumulative Nefopam consumption via IV PCA, for the first 48 h period (mg)	28.0 (17.5–36.9)	35.2 (23.7–41.1)	0.067
PCA clamping, for the first 48 h period	6 (12%)	10 (16.9%)	0.648
Number of patients requiring rescue analgesics for the first 48 h period	15 (30%)	13 (22%)	0.343
Cumulative dose of ketorolac as rescue analgesics for the first 48 h period (mg)	16.8 (32.7)	10.7 (22.1)	0.347
Satisfaction score for pain managements ^a^	77 (67–90)	70 (57–87)	0.140

Data presented as mean (SD), median (interquartile range) or number of patients (proportion). *P* values are the results of unpaired *t*-test or Mann–Whitney test for continuous variables and chi-square test or Fisher’s exact test for incidence variables between the groups. Abbreviations: NRS, numerical rating scale; IV PCA, intravenous patient-controlled analgesia. ^a^ Satisfaction scores for pain managements during the first 48 h postoperative period were assessed with a 101-point numerical rating scale from 0 (totally unsatisfied) to 100 (totally satisfied).

**Table 3:** The analgesic drug-related adverse events.

Outcomes	Predicted Group (*n* = 50)	Control Group (*n* = 59)	*p* Value
Nausea at 3 h postoperatively	9 (18%)	14 (23.7%)	0.465
Nausea at 24 h postoperatively	13 (26%)	20 (33.9%)	0.371
Nausea at 48 h postoperatively	3 (6%)	10 (16.9%)	0.136
Nausea during the 48 h postoperative period	20 (40%)	31 (52.5%)	0.191
Severity of nausea (NRS) ^a^	1.3 (0.7–2.8)	2.3 (1.3–3.0)	0.076
Vomiting	5 (10%)	6 (10.2%)	1.000
Administration of rescue antiemetic agents	5 (10%)	6 (10.2%)	1.000
Respiratory depression	3 (6%)	4 (6.8%)	1.000
Shivering	7 (14%)	6 (10.2%)	0.567
Pruritus	1 (2%)	2 (3.4%)	1.000
Urinary retention	5 (10%)	5 (8.5%)	1.000

Data presented as number of patients (proportion) or median (interquartile range). *P* values are the results of Mann–Whitney *U* test for continuous variables and chi-square test or Fisher’s exact test for incidence variables between the groups. Abbreviations: NRS, numerical rating scale. Postoperative outcomes were evaluated during the first 48 h postoperatively. ^a^ Among the patients who experienced nausea during the 48 hours postoperative period (20 patients in the predicted group; 31 patients in the control group).

## Data Availability

The data sets generated and analyzed during the current study are available on request to the corresponding author upon reasonable request.

## Supplementary Materials

The following are available online at https://www.mdpi.com/2077-0383/10/4/585/s1, Table S1: The subgroup analyses by preoperative pressure pain threshold, Table S2. The subgroup analyses by the types of surgery (laparoscopic, abdominal, and vaginal surgery).
