# Supplementary material for: Predicted Versus Non-Predicted Opioid Administration Using Preoperative Pain Sensitivity in Patients Undergoing Gynecological Surgery: A Randomized-Controlled Trial
Source: J Clin Med. 2021 Feb 4;10(4):585. doi: 10.3390/jcm10040585 (PMC7914520; doi:10.3390/jcm10040585)
Supplement: Supplementary file 1 [file jcm-10-00585-s001.pdf]

Supplementary Materials: Predicted versus Non-Predicted Opioid Administration Using Preoperative Pain Sensitivity in Patients Undergoing Gynecological Surgery: A Randomized-Controlled Trial

Sun-Kyung Park, Hansol Kim, Seokha Yoo, Won Ho Kim, Young-Jin Lim and Jin-Tae Kim

Table S1. The subgroup analyses by preoperative sensitivity to pressure pain.

Outcomes	Sensitive Patients (n = 59) [a]			Non-Sensitive Patients (n = 50) [b]		
	Higher Dose (n = 51)	Lower Dose (n = 8)	p Value	Higher Dose (n = 17)	Lower Dose (n = 33)	p Value
Pressure pain threshold (N)	21.4 (2.7)	21.3 (3.4)	0.946	34.7 (10.8)	37.8 (5.3)	0.278
Pain score at 3 h postoperatively (NRS)	5 (2–7)	4 (3–6.8)	0.902	5 (2.5–8)	5 (2–7)	0.397
Pain score at 24 h postoperatively (NRS)	3 (2–5)	3 (2.3–5.8)	0.831	4 (2.5–5)	3 (2–4)	0.190
Pain score at 48 h postoperatively (NRS)	2 (1–4)	1.5 (1–3)	0.227	3 (1–3.5)	3 (2–3)	0.571
Mean pain score, during the 48-h postoperative period (NRS)	3.7 (2.3–5)	3.2 (2.1–5.2)	0.731	3.7 (2.7–5.5)	3.3 (2.3–4.7)	0.498
Fentanyl consumption within the first 3 h (μg)	93 (69–131)	69 (51–85)	0.101	92 (55–109)	61 (35–98)	0.070
Fentanyl consumption 3–24 h postoperatively (μg)	264 (150–345)	160 (114–189)	0.097	272 (236–368)	203 (141–310)	0.024
Fentanyl consumption 24–48 h postoperatively (μg)	122 (0–347)	159 (19–236)	0.821	290 (0–365)	144 (1.5–237)	0.056
Total cumulative fentanyl consumption for the 48 h period (μg)	501 (320–719)	401 (294–433)	0.156	665 (398–791)	438 (310–505)	0.015
Nausea at 3 h postoperatively	11 (21.6%)	0 (0%)	0.330	6 (35.3%)	6 (18.2%)	0.294
Nausea at 24 h postoperatively	19 (37.3%)	3 (37.5%)	1.000	8 (47.1%)	3 (9.1%)	0.004
Nausea at 48 h postoperatively	7 (13.7%)	1 (12.5%)	1.000	2 (11.8%)	3 (9.1%)	1.000
Nausea during the 48 h postoperative period	28 (54.9%)	3 (37.5%)	0.458	11 (64.7%)	9 (27.3%)	0.015
Vomiting during the 48 h postoperative period	7 (13.7%)	0 (0%)	0.578	1 (5.9%)	3 (9.1%)	1.000

Data presented as mean (standard deviation), median (interquartile range) or number (%). p values are the results of unpaired t-test or Mann-Whitney U test for continuous variables and chi-square test or Fisher's exact test for incidence variables between the groups. Abbreviations: NRS, numerical rating scale. [a] Patients with a pressure pain threshold of < 26 N. [b] Patients with a pressure pain threshold ≥ 26 N.

Table S2. The subgroup analyses by the types of surgery (laparoscopic, abdominal, and vaginal surgery).

Laparoscopic Surgery	Predicted Group (n = 30)	Control Group (n = 40)	p Value
Pressure pain threshold (N)	24 (21–34)	25 (20–33)	0.803
Mean pain score, during the 48 h postoperative period (NRS)	3.3 (1.6)	3.3 (1.6)	0.955

Total cumulative fentanyl consumption for the 48-h period (μg)	341 (251–461)	468 (296–655)	0.060
Nausea during the 48 h postoperative period	11 (37%)	21 (53%)	0.283
Vomiting during the 48 h postoperative period	4 (13%)	4 (10%)	0.957
**Abdominal Surgery**	**Predicted Group (*n* = 11)**	**Control Group (*n* = 12)**	***p* Value**
Pressure pain threshold (N)	23 (20–31)	27 (22–36)	0.449
Mean pain score, during the 48 h postoperative period (NRS)	4.6 (1.8)	5.1 (1.1)	0.385
Total cumulative fentanyl consumption for the 48 h period (μg)	478 (354–784)	714 (572–821)	0.316
Nausea during the 48 h postoperative period	6 (54%)	5 (42%)	0.842
Vomiting during the 48 h postoperative period	0	1 (8%)	1.000
**Vaginal Surgery**	**Predicted Group (*n* = 9)**	**Control Group (*n* = 7)**	***p* Value**
Pressure pain threshold (N)	31 (24–35)	26 (25–34)	0.837
Mean pain score, during the 48 h postoperative period (NRS)	3.2 (1.8)	2.9 (2.0)	0.733
Total cumulative fentanyl consumption for the 48 h period (μg)	492 (409–639)	685 (528–780)	0.408
Nausea during the 48 h postoperative period	3 (33%)	5 (71%)	0.313
Vomiting during the 48 h postoperative period	1 (11%)	1 (14%)	1.000

Data presented as mean (standard deviation), median [interquartile range] or number (%). *p* values are the results of unpaired *t*-test or Mann-Whitney *U* test for continuous variables and chi-square test or Fisher's exact test for incidence variables between the groups. Laparoscopic surgery included laparoscopic ovarian cystectomy, laparoscopic salpingo-oophorectomy, and laparoscopic hysterectomy. Abdominal surgery included abdominal hysterectomy and abdominal myomectomy. Vaginal surgery included vaginal hysterectomy. Abbreviations: NRS, numerical rating scale.
